# Congruency of tumour volume delineated by FET PET and MRSI

**DOI:** 10.1186/2197-7364-2-S1-A61

**Published:** 2015-05-18

**Authors:** Jörg Mauler, Karl-Josef Langen, Andrew A Maudsley, Omid Nikoubashman, Christian Filss, Gabriele Stoffels, N Jon Shah

**Affiliations:** Institute of Neuroscience and Medicine, Forschungszentrum Jülich, Germany; Miller School of Medicine, University of Miami, USA; Department of Neuroradiology, Faculty of Medicine, RWTH Aachen University, Germany

In addition to MR imaging, PET imaging of O-(2-[18F]Fluorethyl)-L-Tyrosine (FET) uptake provides information on brain tumour extent and metabolic activity. Similarly, MRS has been shown to be of value for distinguishing high- from low-grade gliomas. Based on 2D spatially resolved MRSI, an overlap between 18FET uptake and the choline/N-acetyl-aspartate (Cho/NAA) ratio of more than 75 % has been reported.

## Aim

To measure spatial correlation of 18FET-PET with 3D spatially resolved MRSI in patients with gliomas.

## Methods

14 patients (46±16 y) with gliomas (WHO grade II-IV) were examined by simultaneous 18FET-PET-3D-MRSI measurements which covered the whole brain (Siemens BrainPET/3T MR TIM Trio; MRSI: EPSI sequence, TE=17.6 ms). The data were analysed with respect to the congruency of the suspicious tissue delineated by the FET uptake and the Cho/NAA ratio. Locations of individual maxima and distances in between were determined. The congruency of the tumour was assessed using Dice’s coefficients for assumed same tumour volume. All comparisons were carried out at the spatial resolution of the whole brain spectroscopic image (64x64x32 vxl, 5.6x5.6x10mm^3^ each).

## Results

The intensity maxima of both modalities were (61±51) mm distant from each other. The average level of congruency between the tumour volumes delineated in the FET uptake and MRSI data was (33±25)%. Metabolically active tumour tissue, as depicted by FET uptake, is represented to a low extent by the choline/N-acetyl-aspartate ratio measured by spatially resolved 3D MRSI, which is in contrast to previous findings. Both modalities may reflect independent physiological properties of gliomas.

